# COVID-19 Airway Management Isolation Chamber

**DOI:** 10.1177/0194599820942500

**Published:** 2020-07-14

**Authors:** Timothy C. Blood, Jonathan N. Perkins, Paul R. Wistermayer, Joseph S. Krivda, Nathan T. Fisher, Charles A. Riley, Douglas S. Ruhl, Steven S. Hong

**Affiliations:** 1 Department of Otolaryngology–Head and Neck Surgery, Walter Reed National Military Medical Center, Bethesda, Maryland, USA; 2 Department of Otolaryngology–Head and Neck Surgery, Madigan Army Medical Center, Tacoma, Washington, USA; 3 F. Edward Hébert School of Medicine, Uniformed Services University of the Health Sciences, Bethesda, Maryland, USA; 4 Telemedicine and Advanced Technology Research Center, US Army Medical Research and Development Command, Fort Detrick, Maryland, USA; 5 Department of Otolaryngology–Head and Neck Surgery, Fort Belvoir Community Hospital, Fort Belvoir, Virginia, USA; 6 Department of Surgery, Uniformed Services University of the Health Sciences, Bethesda, Maryland, USA

**Keywords:** COVID-19, airborne, aerosolization, airway management, personal protective equipment

## Abstract

**Objective:**

During the coronavirus pandemic (COVID-19), health care workers are innovating patient care and safety measures. Unfortunately, many of these are not properly tested for efficacy. The objective of this study was to determine the efficacy of the novel COVID-19 Airway Management Isolation Chamber (CAMIC) to contain and evacuate particulate.

**Study Design:**

Multi-institutional proof-of-concept study.

**Setting:**

Two academic institutions: Walter Reed National Military Medical Center (WRNMMC) and Madigan Army Medical Center (MAMC).

**Subjects and Methods:**

Smoke, saline nebulizer, and simulated working port models were developed to assess the efficacy of the CAMIC to contain and remove ultrafine particles. Particulate counts were collected at set time intervals inside and outside the system.

**Results:**

With the CAMIC on, smoke particulate counts inside the chamber significantly decreased over time: *r*(18) = −0.88, *P* < .001, WRNMMC; *r*(18) = −0.91, *P* < .001, MAMC. Similarly, saline nebulizer particulate counts inside the chamber significantly decreased over time: *r*(23) = −0.82, *P* < .001, WRNMMC; *r*(23) = −0.70, *P* < .001, MAMC. In the working port model, particulate counts inside the chamber significantly decreased over time: *r*(23) = −0.95, *P* < .001, WRNMMC; *r*(23) = −0.85, *P* < .001, MAMC. No significant leak was detected in the smoke, saline nebulizer, or working port model when the CAMIC was turned on.

**Conclusions:**

The CAMIC system appears to provide a barrier that actively removes particles from within the chamber and limits egress. Further studies are necessary to determine clinical applicability. The CAMIC may serve as an adjunct to improve health care worker safety and patient outcomes.

Severe acute respiratory syndrome coronavirus 2 (SARS-CoV-2) is a virulent and easily transmissible pathogen that causes the novel coronavirus disease 2019 (COVID-19). Health care systems have been overwhelmed due to the highly communicable nature of SARS-CoV-2 and the high morbidity of COVID-19 infection. Although it is primarily transmitted through droplets (via surfaces, hand to face, or close proximity), evolving evidence suggests that it also has aerosol and airborne tendencies.^1-5^ Among patients with severe cases of COVID-19, acute respiratory failure may result in early intubation in lieu of noninvasive ventilation,^3,6^ which places health care workers at a high risk for infection.^7,8^

Mitigation strategies for noninvasive airway management have been proposed, but aerosolization concerns limit their use.^9^ Increased viral concentrations within the nasopharynx^10^ place otolaryngologists at a high risk for exposure^11^ during commonly performed aerosol-generating procedures. Endonasal procedures have been shown to generate significant particulate, which places the provider at risk.^12^ Guidelines to prevent viral propagation during otolaryngologic clinical and surgical treatment recommend donning appropriate personal protective equipment (PPE) based on the procedure being performed.^13^ Health care workers are at high risk for exposure, despite wearing appropriate PPE, as viral particles have been identified in the ventilation systems of hospitals treating patients with COVID-19.^14^ Furthermore, airway interventions present an even higher risk of transmission due to viral aerosolization during intubation^3^ and noninvasive respiratory support mechanisms, including continuous positive airway pressure (CPAP), bilevel positive airway pressure (BiPAP), and high-flow nasal cannula.^2^

In this proof-of-concept study, we introduce the COVID-19 Airway Management Isolation Chamber (CAMIC) system. This functions as a physical barrier and a containment chamber with suction to isolate an infected patient. We hypothesize that the CAMIC will contain and evacuate particles within the system, with limited egress into the surrounding environment. The primary outcome will examine the reduction in particulates over time through the CAMIC system. The secondary outcome will evaluate particulate escape from the CAMIC system.

## Methods

### CAMIC Assembly

**Figure 1.**
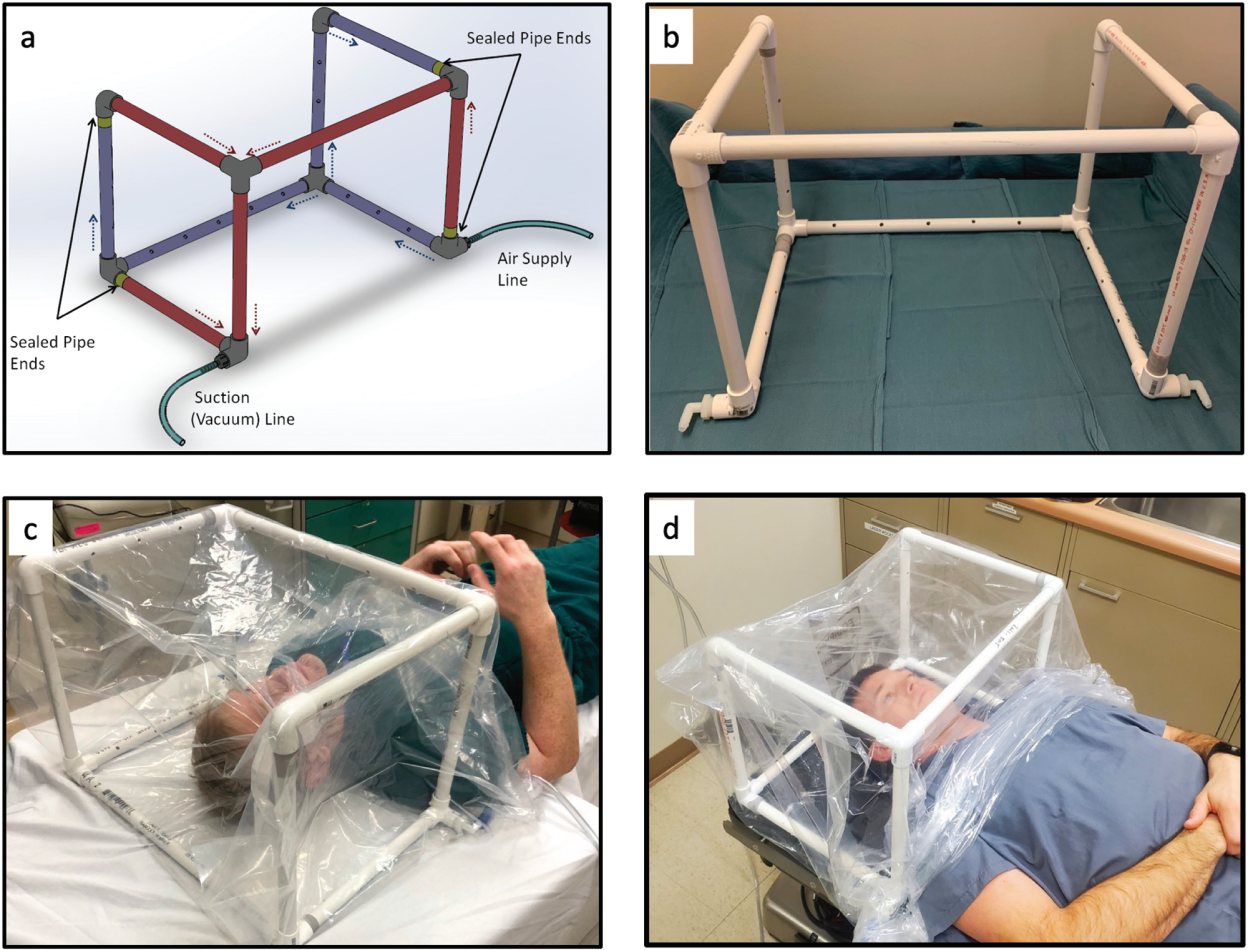
(a) CAMIC frame drawing: red, suction outflow; blue, air inflow; yellow, occluded suction and air flow. (b) Assembled CAMIC frame. (c) Superior view. (d) Inferior view. CAMIC, COVID-19 Airway Management Isolation Chamber.

The CAMIC system is a polyvinyl chloride hollow frame with fenestrations. It is placed at the head of a hospital or surgical bed (**Figure 1**). Its assembly is described in Supplement 1 (available online). Suction is attached and distributed via the frame. Oxygen is delivered through the contralateral port. The CAMIC system is inserted into a clear surgical bag (40 × 40 in, SYP404025CL, Medline Industries, Walter Reed National Military Medical Center [WRNMMC]; 28 × 22 × 54 in, 81-1102, Tri-Anim Health Services, Madigan Army Medical Center [MAMC]) and placed around the head, neck, and shoulders of the patient. At WRNMMC, the barrier covering included a drawstring that was cinched around the mannequin’s torso, whereas at MAMC, the barrier had no drawstring and was tucked tightly around the mannequin’s torso.

### Testing Conditions

Testing was completed independently at 2 academic medical centers, WRNMMC and MAMC, within standard clinic procedure rooms that were similar in size and configuration and not HEPA-filtered (high-efficiency particulate air) negative-pressure rooms. For the smoke and nebulizer models, particle readings were taken inside (center of the chamber) and outside (3 in from the bag on the torso of the mannequin) of the CAMIC system at set intervals. The particle detector remained in a constant position on the mannequin’s torso during testing. An internal sample line was suspended in the middle of the CAMIC above the mannequin’s face and run externally, which allowed internal measures without placement of the particle detector within the CAMIC. The sample line was capped to prevent undue particle egress. For the working port model, particle readings were taken 1 in from a 4-in slit in the superior panel of the CAMIC. An internal sample line suspended above the mannequin’s head allowed for internal measurements.

**Figure 2.**
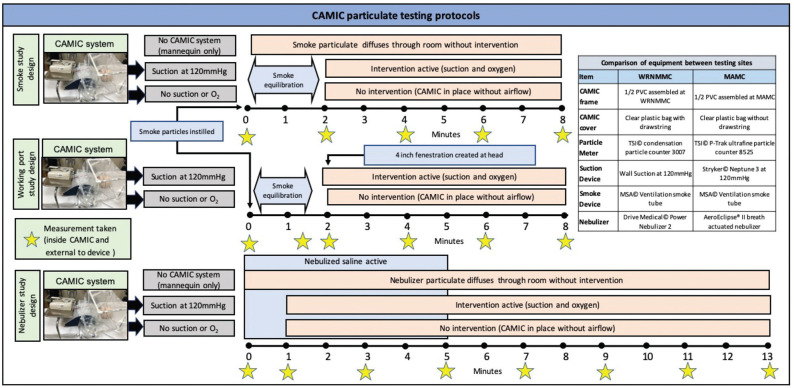
CAMIC particulate testing methodology. CAMIC, COVID-19 Airway Management Isolation Chamber; MAMC, Madigan Army Medical Center; WRNMMC, Walter Reed National Military Medical Center.

Industrial-grade condensation particle counters (WRNMMC, model 3007, TSI Incorporated; MAMC, model 8525, P-Trak Ultrafine Particle Counter) collected particle counts with 10-second means. The 2 particle meters show good correlation on comparative evaluations.^15^ A smoke model approximated ultrafine particles (0.5 µm), while a saline nebulizer model approximated microdroplets/aerosol particles (0.5-5 µm). A working port model assessed the effectiveness of the CAMIC with a deliberate fenestration into the barrier. Each scenario was tested with the suction and oxygen on within the CAMIC and without any suction or oxygen within the CAMIC. Five iterations of each scenario were conducted at both institutions. Background readings were taken inside and outside the CAMIC prior to each trial for reference. The particle detector was kept in the same location for each testing scenario. Negative controls were recorded to establish particle dissipation for each test room via the same testing methods as in the smoke model with a CAMIC without the polyethylene barrier, suction, or airflow (**Figure 2**).

Institutional Review Board approval was obtained at both institutions (WRNMMC-EDO-2020-0471, 925444; MAMC-20-09739).

### Smoke Particulate Testing

A ventilation smoke tube (MSA Safety Incorporated) containing ethylenediamine and acetic acid delivered 6 puffs of smoke within the CAMIC directly above the mannequin’s face. Six puffs of smoke were used to produce a replicable high particulate count within the CAMIC system. A 2-minute period was used to allow for adequate smoke distribution within the CAMIC. Initial particle counts were recorded. Suction (120 mm Hg) and oxygen (10 L/min) were started after 2 minutes of equilibration. Internal and external particle counts were recorded every 2 minutes over an 8-minute period, which was chosen after initial testing where 8 minutes were required to evacuate particulate near external environment baseline.

### Nebulizer Particulate Testing

Power Nebulizer 2 (WRNMMC; Drive Medical) or AeroEclipse II Breath Actuated (MAMC; Monaghan Medical) nebulized normal saline within the CAMIC for 1 minute to allow for adequate particle distribution. At WRNMMC the nebulized normal saline was delivered via a face mask attached to the mannequin, whereas at MAMC the mouthpiece was secured to the mannequin’s mouth. An initial particulate count was recorded. Suction (120 mm Hg) and oxygen (10 L/min) were started. The nebulizer was run for 4 additional minutes to simulate a nebulizer treatment. Internal and external particle counts were recorded every 2 minutes over a total of 12 minutes, which was 8 minutes after cessation of nebulization.

### Working Port Testing

Our smoke model methodology was modified to simulate a working port or a 4-in vertical slit (where a provider managing the airway would stand) with the CAMIC. After application of 6 puffs of smoke within the CAMIC, a peak reading was taken at 90 seconds before creation of a working port. Wall suction (120 mm Hg) and oxygen (10 L/min) were started at 2 minutes. Internal and external particle counts were recorded every 2 minutes over an 8-minute period.

### Statistical Analysis

Particulate counts were analyzed with Microsoft Excel. Descriptive statistics were reported. Paired *t* testing and Pearson’s correlation coefficient assessed the relationship between particle counts within trials over time. For the smoke and working port models, the 2-minute and 90-second readings were used as the reference for intratest comparisons. For the nebulizer model, intratest comparisons were performed over the first 5 minutes when the nebulizer was running and for the subsequent 8 minutes after discontinuation of nebulizer therapy. Student’s *t* testing was performed to compare treatment versus control at each time point. A power analysis was performed; 95% CIs were reported; and statistical significance was determined to be *P* < .05. Background readings were removed for all statistical analyses.

## Results

### Smoke Particulate Testing

**Figure 3.**
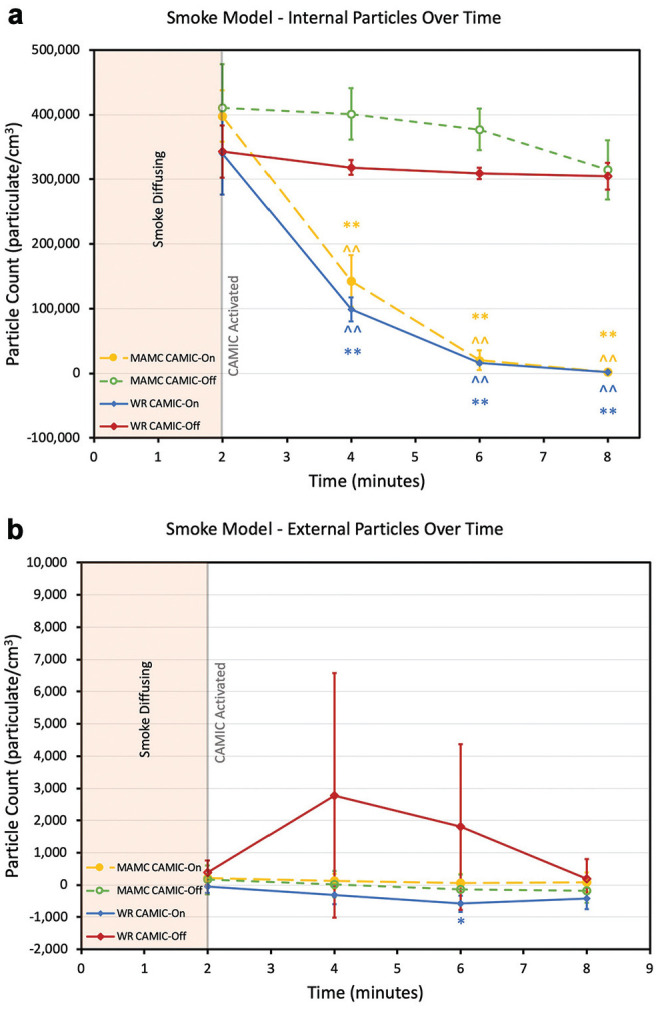
Smoke particulate model: (a) internal and (b) external particles over time. Gray line, CAMIC activation. Mean and 95% CI are presented. Intertest comparisons between CAMIC-On and CAMIC-Off: ^*P* < .05. ^^*P* < .001. Intratest comparisons: *P < .05. **P < .001. CAMIC, COVID-19 Airway Management Isolation Chamber; MAMC, Madigan Army Medical Center; WRNMMC, Walter Reed National Military Medical Center.

Internal particle counts decreased at each time point at both institutions with CAMIC-On (**Figure 3a**). Particulate counts within the chamber for the CAMIC-On models negatively correlated to time at both institutions: *r*(18) = −0.88, *P* < .001, WRNMMC; *r*(18) = −0.91, *P* < .001, MAMC. There was no significant decrease in internal particle count over time in the CAMIC-Off trials. At both institutions, the internal particle count was significantly lower with CAMIC-On versus CAMIC-Off at 4, 6, and 8 minutes.

External particle counts were significantly lower with CAMIC-On and CAMIC-Off as compared with negative controls at both institutions (except at WRNMMC, 6 and 8 minutes with CAMIC-Off and CAMIC-On; Supplemental Table S1). No evidence of external particle leak was detected for the CAMIC-On or CAMIC-Off scenarios at either institution (**Figure 3b**).

### Nebulizer Particulate Testing

**Figure 4.**
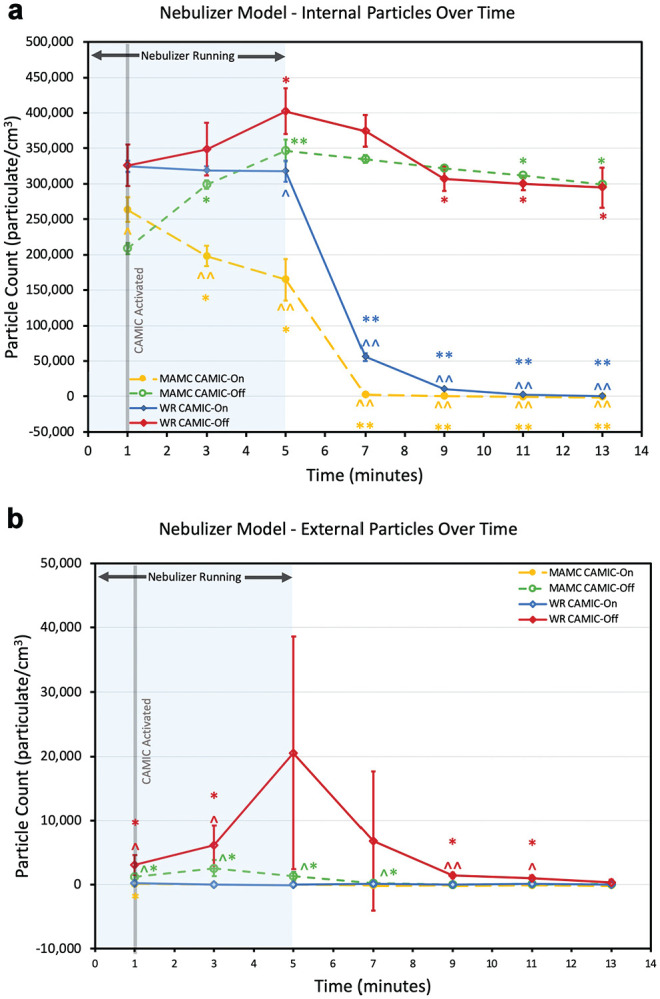
Nebulizer particulate model: (a) internal and (b) external particles over time. Gray line, CAMIC activation. Mean and 95% CI are presented. Intertest comparisons between CAMIC-On and CAMIC-Off: ^*P* < .05. ^^*P* < .001. Intratest comparisons: *P < .05. **P < .001. CAMIC, COVID-19 Airway Management Isolation Chamber; MAMC, Madigan Army Medical Center; WRNMMC, Walter Reed National Military Medical Center.

During the nebulizer treatment, internal particle counts significantly decreased at MAMC at 3 and 5 minutes. After the cessation of nebulizer treatment, internal particle counts decreased at all time points (7, 9, 11, and 13 minutes) at both institutions with CAMIC-On (**Figure 4a**). The CAMIC-On internal nebulizer particulate counts negatively correlated to time at both institutions: *r*(23) = −0.82, *P* < .001, WRNMMC; *r*(23) = −0.70, *P* < .001, MAMC. There was a significant decrease in internal particle count over time in the CAMIC-Off trials at the 11- and 13-minute time points at MAMC and the 9-, 11-, and 13-minute time points at WRNMMC. At both institutions, the internal particle count was significantly lower with CAMIC-On versus CAMIC-Off at all time points following the cessation of nebulizer treatment.

External particle counts were significantly lower with CAMIC-On and CAMIC-Off as compared with negative controls at all time points at both institutions (except WRNMMC, 11 and 13 minutes with CAMIC-Off and 13 minutes with CAMIC-On; Supplemental Table S2). No evidence of external particle leak was detected for the CAMIC-On scenarios at either institution (**Figure 4b**).

### Working Port Testing

**Figure 5.**
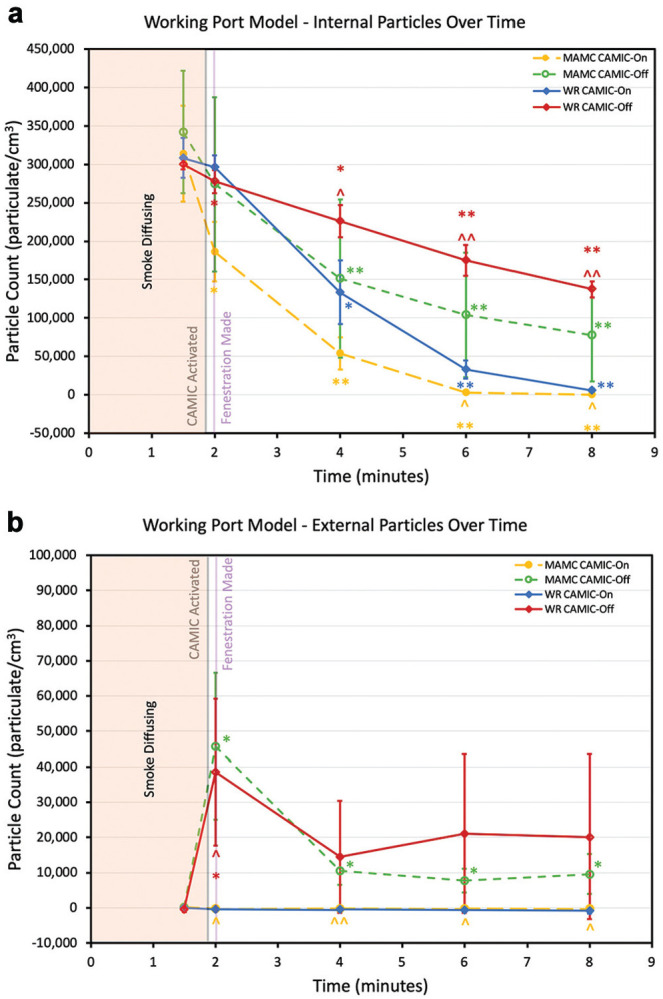
Working port model: (a) internal and (b) external particles over time. Gray line, CAMIC activation. Purple line, fenestration creation. Mean and 95% CI are presented. Intertest comparisons between CAMIC-On and CAMIC-Off: ^*P* < .05. ^^*P* < .001. Intratest comparisons: *P < .05. **P < .001. CAMIC, COVID-19 Airway Management Isolation Chamber; MAMC, Madigan Army Medical Center; WRNMMC, Walter Reed National Military Medical Center.

After the working port was created, internal particle counts significantly decreased at each institution at all time points with CAMIC-On and CAMIC-Off (**Figure 5a**). CAMIC-On particle counts negatively correlated to time at both institutions: *r*(23) = −0.95, *P* < .001, WRNMMC; *r*(23) = −0.85, *P* < .001, MAMC. Internal particle counts significantly decreased over time after creation of the working port in the CAMIC-Off scenarios at each institution. CAMIC-Off particle counts negatively correlated to time at both institutions: *r*(23) = −0.96, *P* < .001, WRNMMC; *r*(23) = −0.7, *P* < .001, MAMC. Internal particle counts were significantly lower with CAMIC-On as compared with CAMIC-Off at 4, 6, and 8 minutes at WRNMMC and 6 and 8 minutes at MAMC.

After creation of the working port, no significant increase in external particle count was detected at either institution with CAMIC-On. With CAMIC-Off, a significant increase in external particle count was observed at both institutions following creation of the working port (at the 2-minute mark). Elevated external particle counts (CAMIC-Off vs CAMIC-On) were detected at 4, 6, and 8 minutes at MAMC.

## Discussion

The COVID-19 pandemic has tested the limits of health care systems around the world. Airborne spread,^1-5^ asymptomatic carriers,^16^ viral droplets that can remain on surfaces for several days,^17^ and inaccurate patient testing^18^ all contribute to its high transmission rates. Critically ill patients requiring mechanical ventilation often succumb to a deadly pulmonary illness^19-21^ with features atypical to acute respiratory distress syndrome.^22^ Initial concerns regarding aerosolization of the virus prompted deviation from standard critical care airway algorithms, limiting the use of noninvasive therapy such as CPAP and BiPAP.^23,24^ These guidelines recommend early intubation for definitive airway management,^25,26^ which has downstream effects on the availability of ventilators. Retrospective data from China and Italy suggest that hypoxemic patients with COVID-19 respond well to positive pressure, indicating a crucial role for noninvasive respiratory treatment as an alternative to intubation.^27^ Our aim in development of the CAMIC was to create a multifunctional adjunct PPE barrier to protect health care workers intervening on the airway in various capacities. In this proof-of-concept study, we designed and tested a novel isolation chamber, the CAMIC, which has the potential to serve 3 vital functions: (1) it provides a resilient barrier between patients and health care workers; (2) it facilitates a safer environment for noninvasive airway management; and (3) it is assembled with readily available equipment.

Previous barrier methods appear to have significant limitations. In Italy, a self-contained helmet was used with the intent to reduce intubation rates and extend the benefits of noninvasive ventilation.^28^ However, intubation is not possible through this system, and clearance of particulates has not been adequately assessed. Another barrier, the rigid aerosol box, may decrease droplet exposure, though droplets may escape the working ports.^9^ In addition, the open end of the box allows respiratory particles to escape into the room.^29^ In the otolaryngology literature, endonasal drilling and transnasal cauterization have been shown to produce significant particulate, which places providers at risk for exposure in the absence of using N95 respirators.^12^ Other novel systems have been used for otolaryngologic aerosol-generating procedures, including a flexible barrier for otologic procedures^30^ and a negative-pressure system for performing tracheostomy.^31^ While these reports demonstrate the clinical feasibility of these systems, all are based on anecdotal evidence. In contrast, we have demonstrated that the CAMIC contains and removes particulate with reproducible efficacy.

The CAMIC provides a droplet barrier but also uses an internal vacuum and oxygen system, which may create laminar flow that facilitates particulate removal.^32^ Our data demonstrate that the CAMIC contained and removed >99% of smoke and nebulized saline particles introduced within the system, with minimal leaks into the environment. This is particularly notable in the nebulizer model, where particle counts decreased despite active nebulization within the closed CAMIC system. When a working port was introduced, the CAMIC prevented a detectable leak when turned on. While dynamic testing is beyond the scope of this proof-of-concept study, these findings encourage future investigations to use the CAMIC as a device for particulate or droplet containment and evacuation during active respiratory interventions. Furthermore, the CAMIC could function as an additional PPE barrier during otolaryngologic interventions, such as flexible and direct laryngoscopy, nasal endoscopy, and sinus surgery, as well as otologic surgery.

The 2 institutions performed testing by using different nebulizers and polyethylene bags to confirm efficacy with commonly available hospital resources. This variability demonstrated that results are reproducible. The simple design and easily accessible materials allow the CAMIC to be assembled without specialized equipment. This device could be useful in a variety of clinical settings and has the potential for mass production.

This was a small-sample study to demonstrate proof of concept. Other limitations include variability in test room conditions, such as differences in air turnover in the MAMC and WRNMMC procedure rooms, as well as the use of a mannequin. Control testing of the procedure rooms at MAMC and WRNMMC demonstrated different rates of smoke dissipation, suggesting differences in the HVAC systems (heating, ventilation, and air conditioning) at each institution (Supplemental Table S1). Additionally, we were able to conduct particle measurements only at set time points and locations.

In preliminary testing, we found that the smoke distributed within the CAMIC within 1 to 2 minutes, whereas the nebulized saline distributed at approximately 1 minute, which is why the CAMIC was activated at different time points in these models. An 8-minute period was chosen for our smoke model because in our initial testing, this was the time required to evacuate particulate near baseline. However, in the nebulizer model, after the 1-minute initial equilibration, we ran the nebulizer for an additional 4 minutes to simulate a nebulization treatment. Similar to the smoke model, measurements were taken for an additional 8 minutes, yielding a total nebulizer trial of 13 minutes. In our working port model, the peak measurement was taken at 90 seconds to allow for peak particle concentration capture, slit (working port) creation, and then immediate capture of the potential leak. The additional 30 seconds allowed for performance of the additional tasks and reading capture.

The decrease in particle counts in the nebulizer model at the 11- and 13-minute time points at MAMC and the 9-, 11-, and 13-minute time points at WRNMMC may be due to settling of the larger droplets to the bottom of the chamber. Both institutions had similar results, but significance may be due to using 2 different nebulizers and particle counters. Similar to our smoke external data as compared with the no-CAMIC control model, we attribute the differences seen in Supplemental Table S2 to the HVAC air turnover within the procedure rooms at MAMC and WRNMMC.

Future studies should incorporate continuous measurements from multiple locations when assessing dynamic treatments and aerosol-generating procedures, such as laryngoscopy, endoscopic sinus surgery, mastoidectomy, and tracheostomy. These should examine the applicability of intubation, patient transport, and surgical intervention. While we demonstrated that the CAMIC significantly reduced particulate count, clinical effectiveness warrants future study.

## Conclusion

The CAMIC appears to provide a barrier that actively removes particles from within the chamber and limits egress into the treatment room. This could allow for safer noninvasive airway management, such as high-flow oxygen, nebulizers, and CPAP/BiPAP. The device evacuates internal particulate, even with a working port, suggesting procedural utility during invasive airway management. Furthermore, the CAMIC is easily assembled with readily available inexpensive materials. The CAMIC does not replace the need for individual PPE but may serve as an adjunct to improve health care worker safety and patient outcomes.

## Supplemental Material

CAMIC_appendix_build_design_final05032020 – Supplemental material for COVID-19 Airway Management Isolation Chamber Click here for additional data file. Supplemental material, CAMIC_appendix_build_design_final05032020 for COVID-19 Airway Management Isolation Chamber by Timothy C. Blood, Jonathan N. Perkins, Paul R. Wistermayer, Joseph S. Krivda, Nathan T. Fisher, Charles A. Riley, Douglas S. Ruhl and Steven S. Hong in Otolaryngology–Head and Neck Surgery

## Supplemental Material

E-Tables_Revised – Supplemental material for COVID-19 Airway Management Isolation Chamber Click here for additional data file. Supplemental material, E-Tables_Revised for COVID-19 Airway Management Isolation Chamber by Timothy C. Blood, Jonathan N. Perkins, Paul R. Wistermayer, Joseph S. Krivda, Nathan T. Fisher, Charles A. Riley, Douglas S. Ruhl and Steven S. Hong in Otolaryngology–Head and Neck Surgery
