# Supplementary material for: COVID-19 Airway Management Isolation Chamber
Source: Otolaryngol Head Neck Surg. 2020 Jul 14:0194599820942500. doi: 10.1177/0194599820942500 (PMC7361124; doi:10.1177/0194599820942500)

**COVID-19 Airway Management Isolation Chamber (CAMIC)
Assembly Instructions**

**DISCLAIMER:**

**The instructions below are to assist with assembling the COVID-19 Airway Isolation Management Chamber (CAMIC). The CAMIC is an adjunctive protective barrier designed to mitigate risk to health care workers (HCW). The CAMIC is not meant to be a stand-alone unit of protective equipment. The CAMIC should always be used with approved personal protective equipment and pursuant to the guidance of your institution. All connections should be secured and checked frequently. Anytime anyone is within the CAMIC, direct observation is required.**

**An emergency use authorization (EUA) has been granted from the U.S. Food & Drug Administration (#PEUA200438). This device has two provisional patents on behalf of the U.S. Department of Defense (#63/008,033) submitted 10 April, 2020 and (#63/009885) submitted 14 April, 2020.**

Materials Needed:

- ½ inch schedule 40 PVC pipe - 11 feet total length cut in sections:
  - 8- 12 inch length PVC pipe sections
  - 2- 18 inch length PVC pipe sections
- 4- 1/2 inch schedule 40 PVC 90° elbow with a side outlet with slip (female socket) x slip x slip connections
- 2- 1/2 inch schedule 40 PVC 90° elbow with a side outlet with slip x slip x thread connections
- 2- 3/8 inch I.D. x 1/2 in. NPT plastic barbed tube fitting, 90° elbow
- 1- Surgical glove
- 1- Large clear plastic bag (preferably with drawstring- 40”x40”)

Tools Required:

- ¼ inch drill bit with electric drill
- Cutting tool (Hacksaw or PVC cutters)
- Tape measure

**Figure 1: Materials**


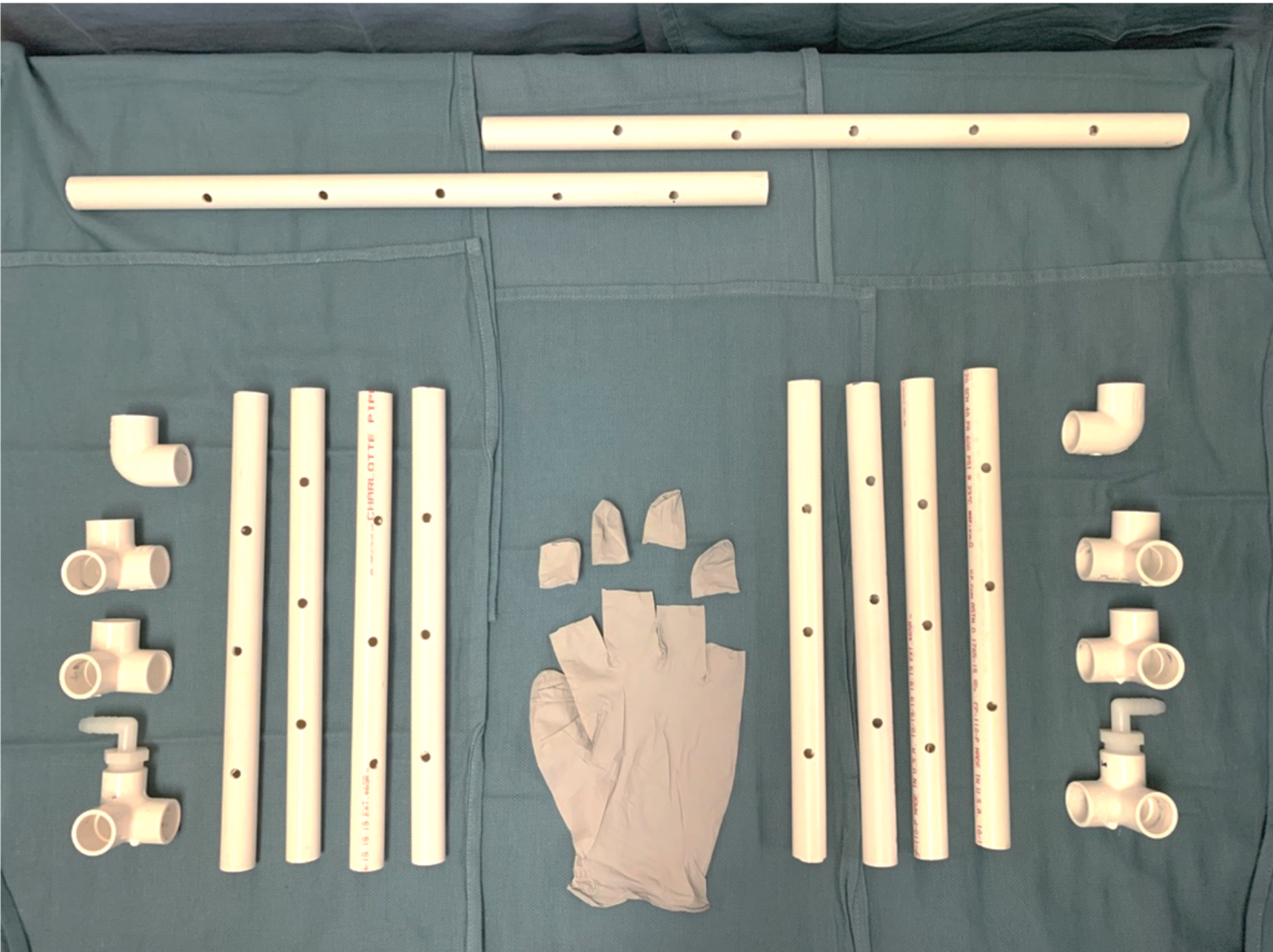


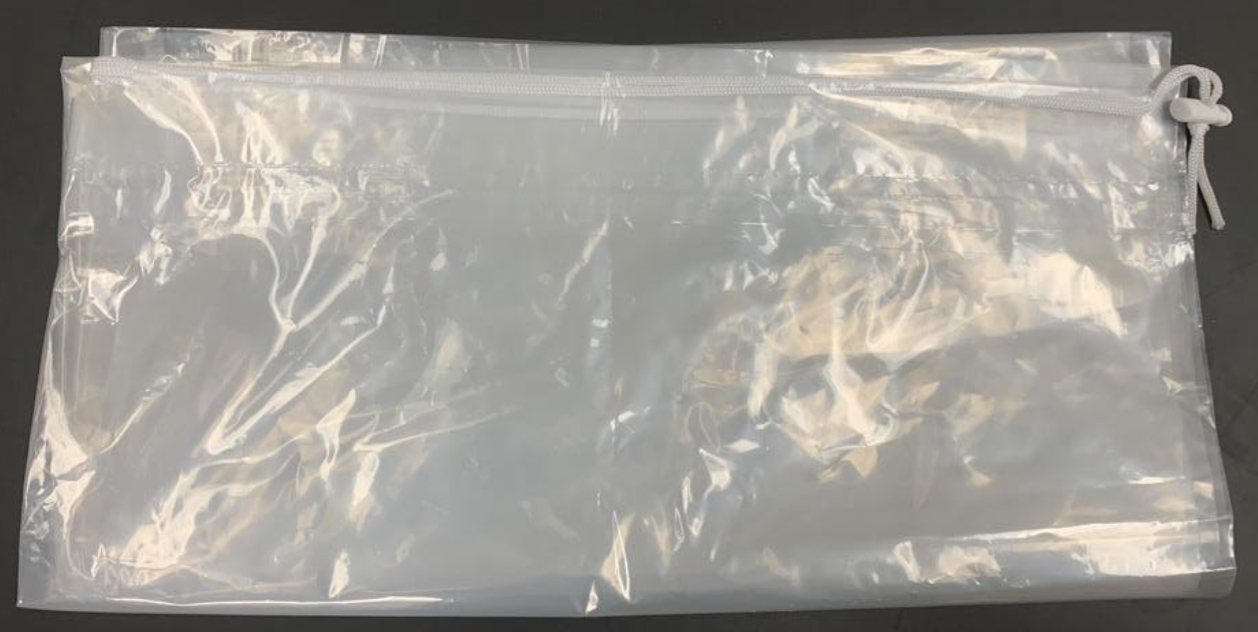


Assembly Instructions

**Preparing Pipe Sections:**

- 1. Cut ½ inch schedule 40 PVC pipes to size (2-18”L, 8-12”L)
  2. Cut 4 fingertips off surgical glove (Fig. 2)
  3. Drill suction/air ports
     1. Drill one ¼” hole at pipe mid-length, through one surface only and perpendicular to the pipe central axis.
     2. For 12”L Sections: Drill 2 additional ¼” holes measured 3” from the first hole center, in both directions along the pipe central axis such that all 3 hole axes are coplanar.
     3. For 12”L Sections: Drill 4 additional ¼” holes measured at 3” and 6” from the first hole center, in both directions along the pipe central axis such that all 5 hole axes are coplanar.
  4. Seal the end of 4 separate 12”L pipe sections by stretching 4 of the cut surgical glove fingertips over one end the pipe (Figure 2)

**Figure 2: Sealing end of pipe section**


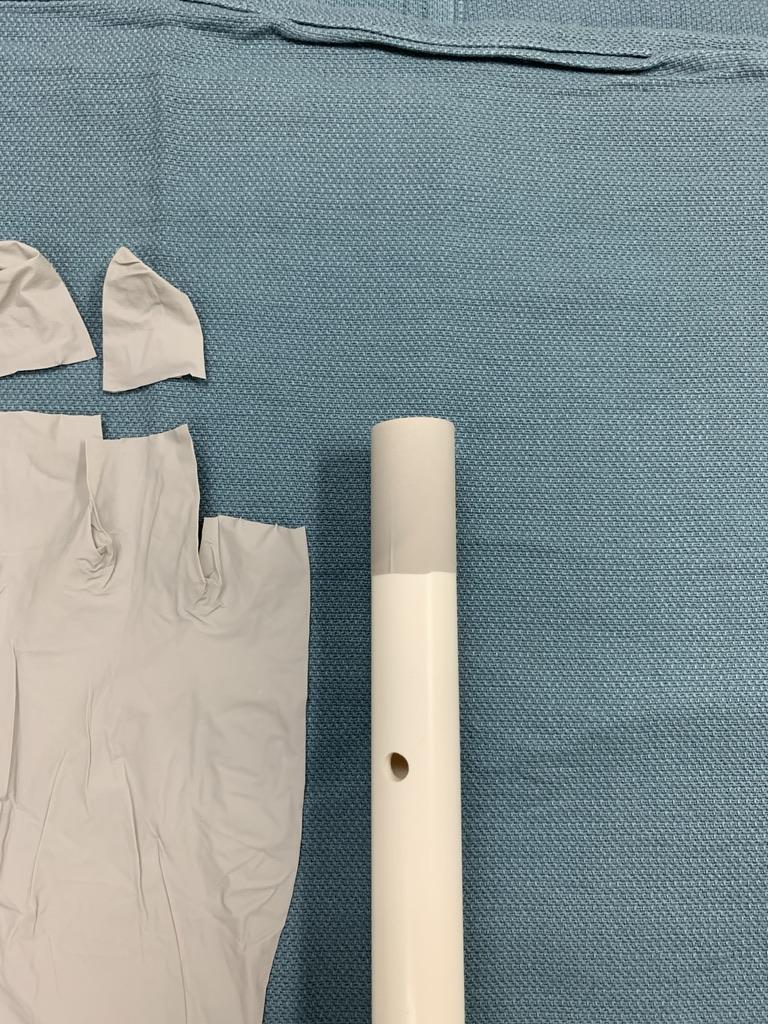


**Assembling Frame:**

- 1. Using the 12”L sections, assemble the square outer frames shown below (Figure 3) by firmly pressing pipe sections into pipe connectors. Ensure location of the sealed ends of the pipe lengths match the configuration shown. For each pipe, orient the drilled holes such that the plane containing the drilled hole axes are oriented 45° to the plane containing the central axes of the adjacent pipe frame members.

**Figure 3: Configuration of outer square frames, built from 12”L Sections**


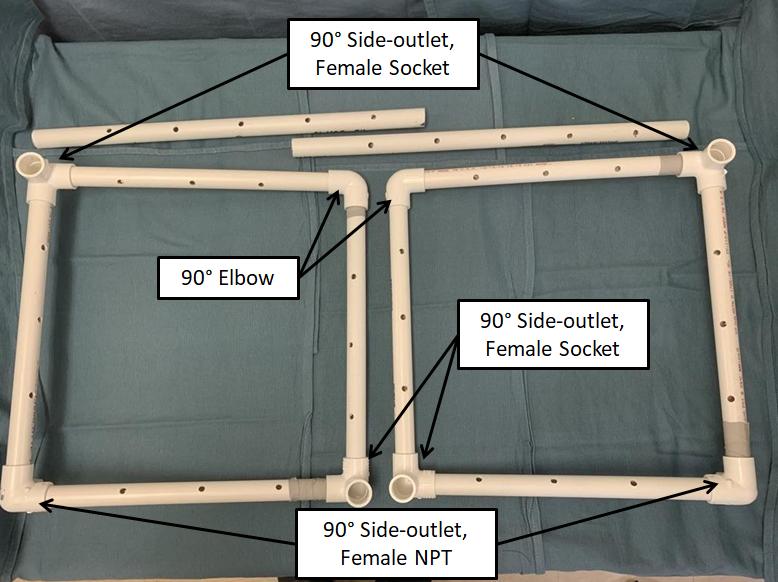


- 1. Join square outer frames by connecting the 18”L pipe sections as shown in Figure 4. For each pipe, orient the drilled holes such that the plane containing the drilled hole axes are oriented 45° to the plane containing the central axes of the adjacent pipe frame members.
  2. Screw 2 plastic barbed tube fittings into threaded side ports as shown in Figure 4.

**Figure 4: Outer square frames joined with inner 18”L pipe sections and tube fittings connected**


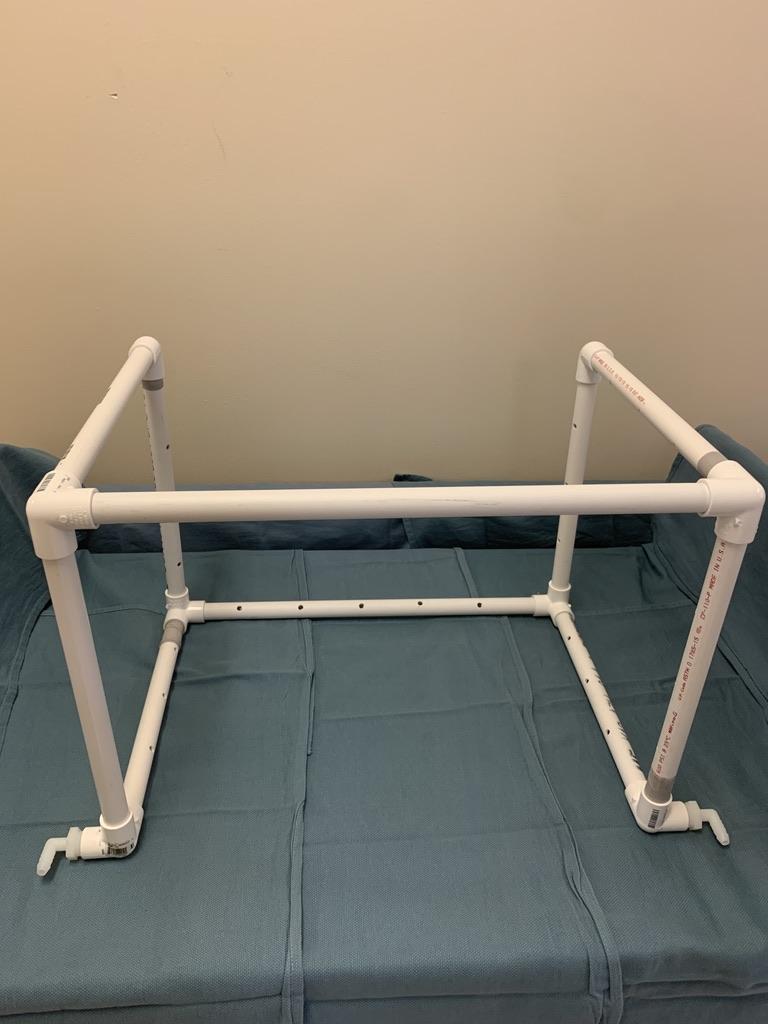


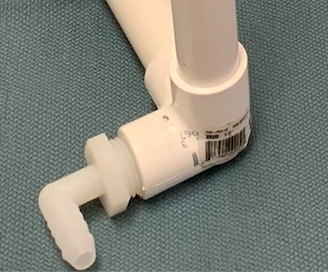


**Airflow through Frame:**

- 1. Confirm placement of sealed pipe ends to direct airflow appropriately (Figure 5):
- Blue= medical air or oxygen
- Red= suction
- Yellow= airflow blocked at pipe end by surgical glove fingertip sleeve over pipe
  1. Attach suction line to blue side (at least 120 mmhg)
  2. Attach air supply line (medical air or oxygen) at least 10L/min flow rate

**Figure 5: Airflow direction through frame**


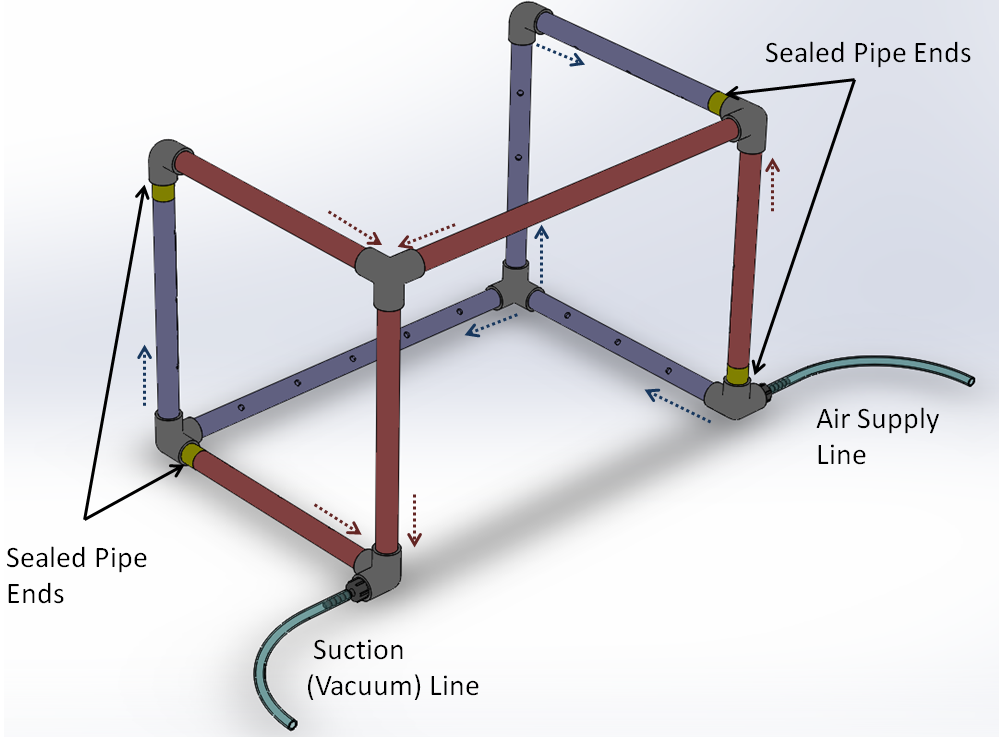


**Final Assembly:**

- 1. Place large clear bag with drawstring over frame (40x40” clear bag with drawstring). Orient the bag such that the drawstring can be cinched around the shoulders of the patient (Figure 6).

**Figure 6: Final assembly, clear bag over frame**


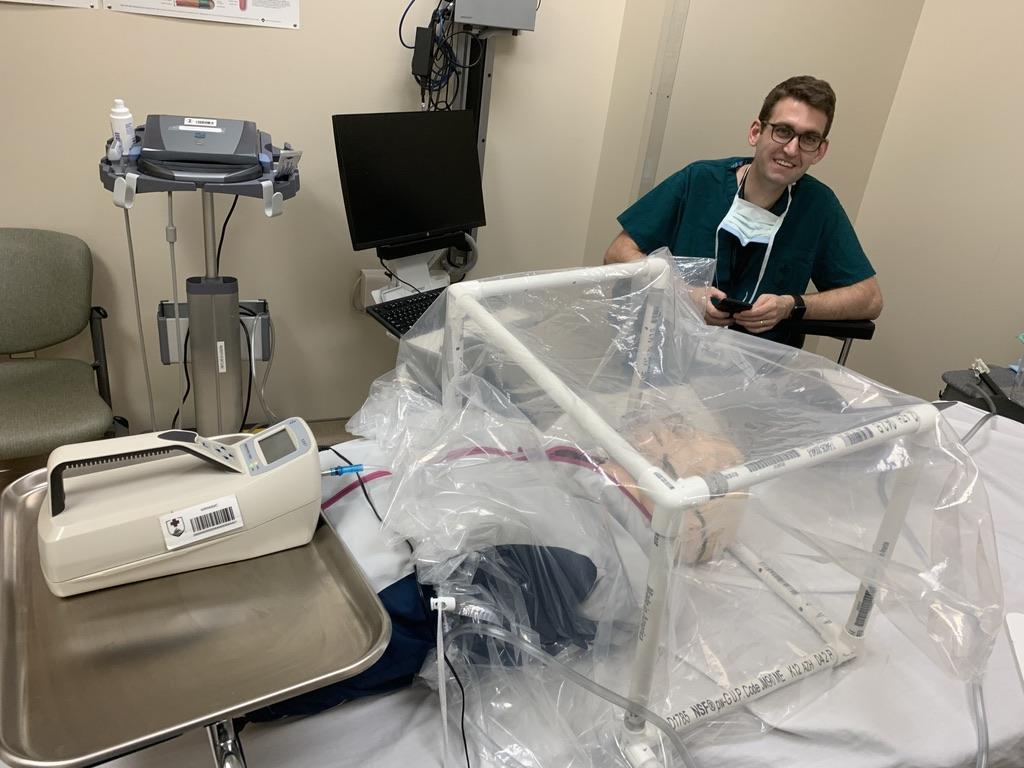


**Additional Diagrams:**

**Figure 7: Exploded view of frame assembly**


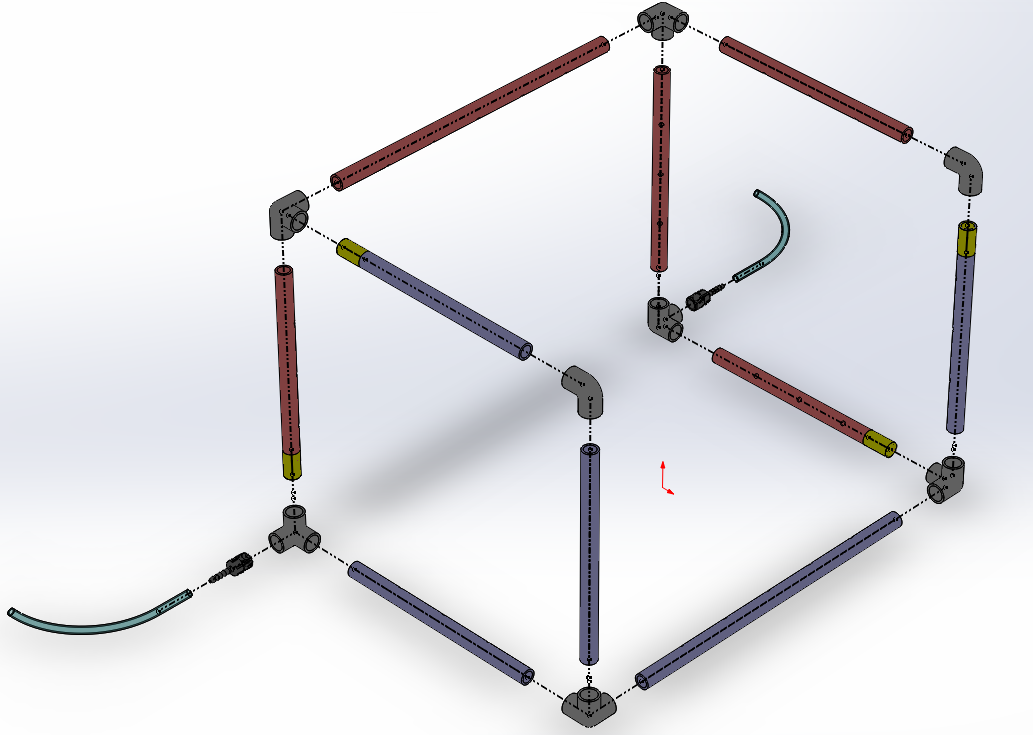


**Figure 8: View of completed frame assembly**


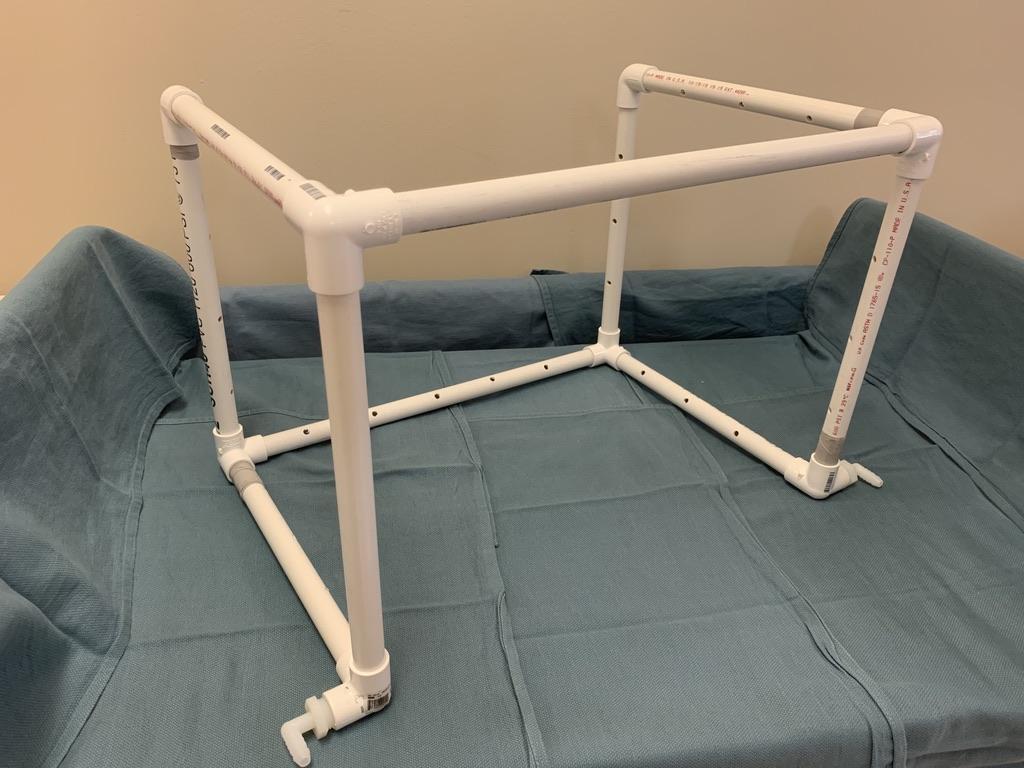


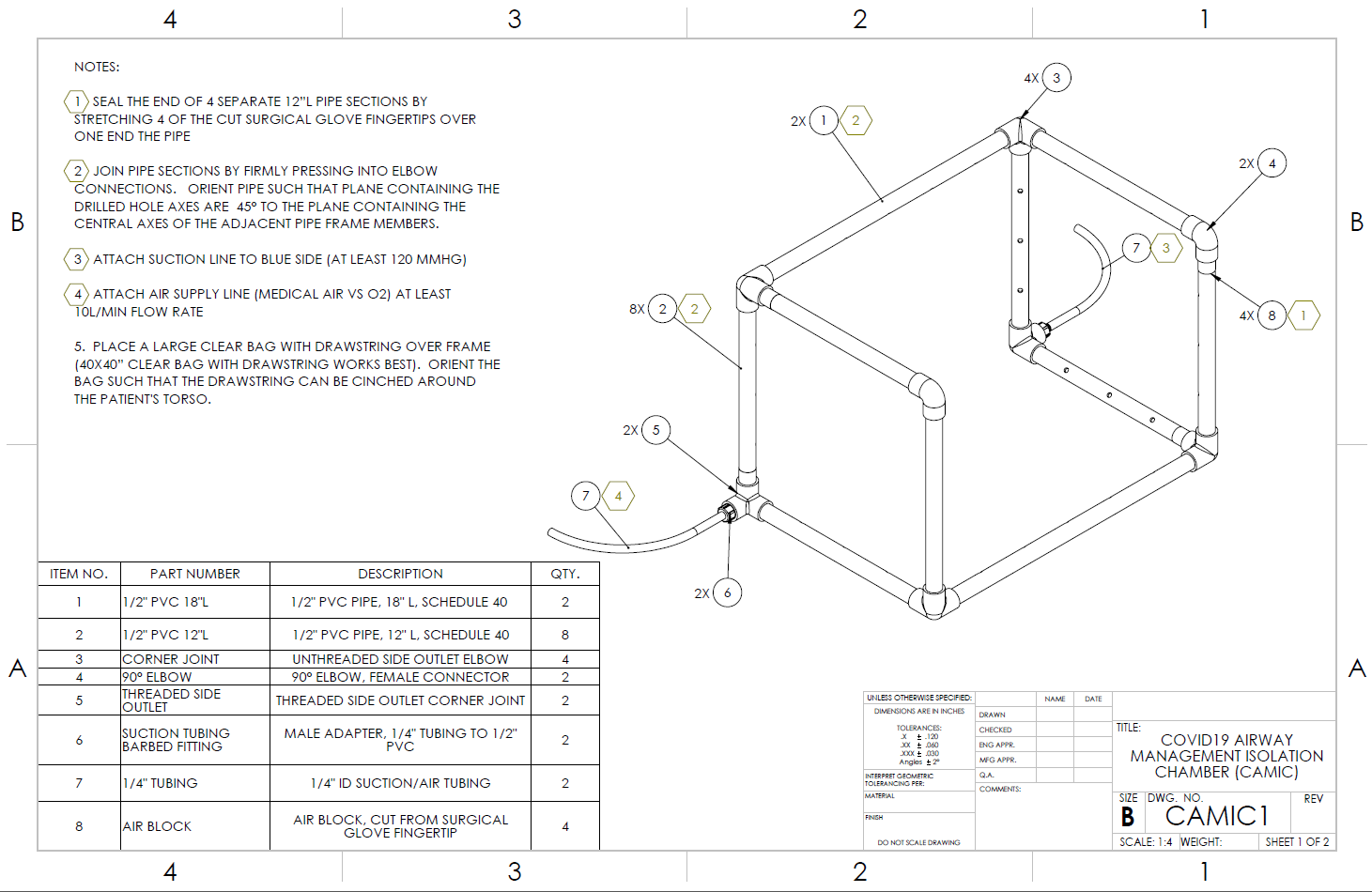


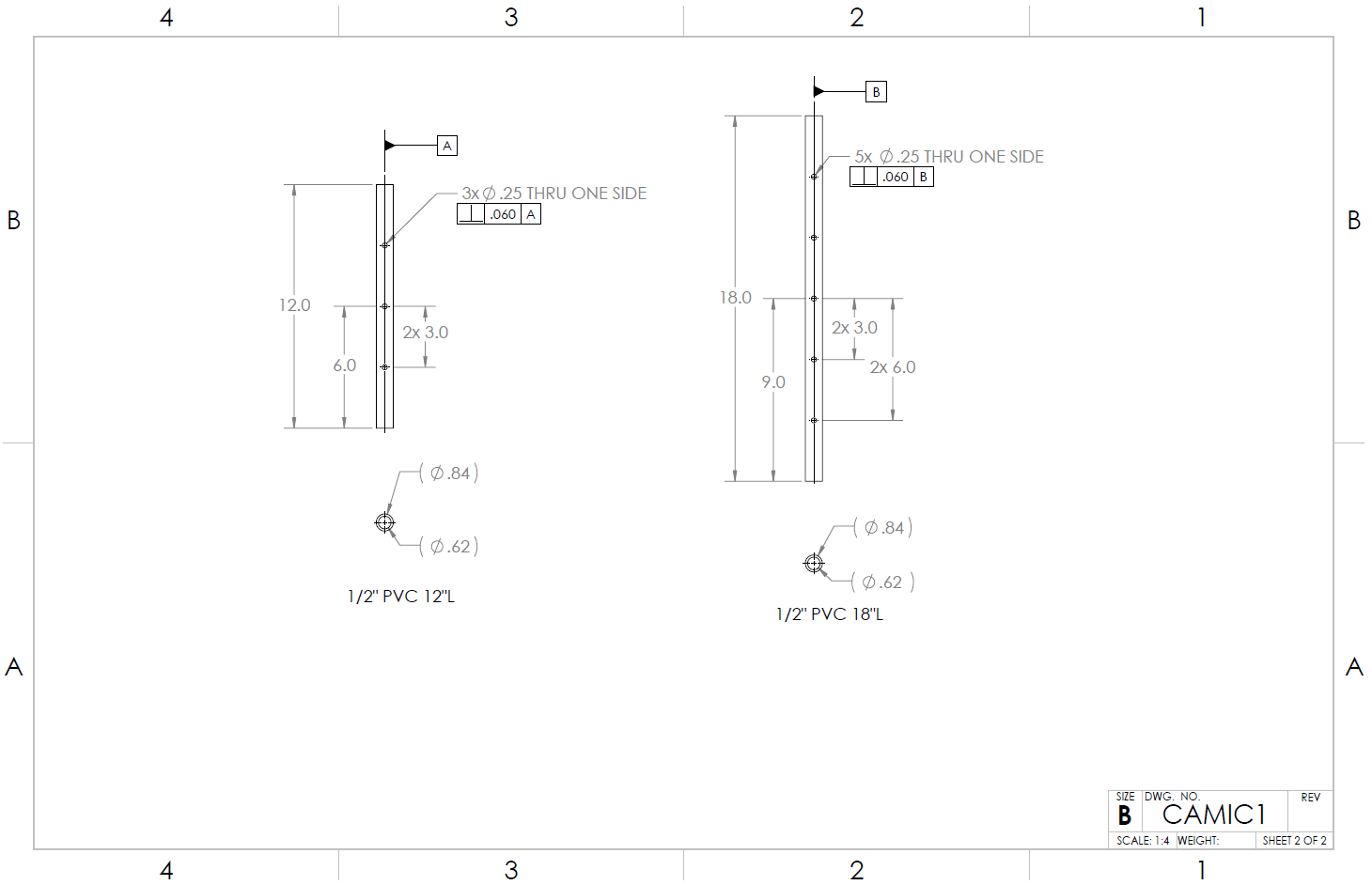

Supplement: CAMIC_appendix_build_design_final05032020 – Supplemental material for COVID-19 Airway Management Isolation Chamber [file CAMIC_appendix_build_design_final05032020.docx]
