# Supplementary material for: COVID-19 Airway Management Isolation Chamber
Source: Otolaryngol Head Neck Surg. 2020 Jul 14:0194599820942500. doi: 10.1177/0194599820942500 (PMC7361124; doi:10.1177/0194599820942500)
Supplement: E-Tables_Revised – Supplemental material for COVID-19 Airway Management Isolation Chamber [file E-Tables_Revised.docx]

**eTable 1. Particulate counts for smoke model outside CAMIC system**

| All values particulate/cm3 | **2 minutes** | | **4 minutes** | | **6 minutes** | | **8 minutes** | |
| --- | --- | --- | --- | --- | --- | --- | --- | --- |
|  | Mean | SD | Mean | SD | Mean | SD | Mean | SD |
| **WR Control (No CAMIC)** | 2500 | 529 | 1333 | 702 | 400 | 954 | -133 | 751 |
| **WR CAMIC on** | -60 | 207 | -320 | 303 | -580 | 286 | -420 | 383 |
| **t-test (vs control)** | <.001 | | .003 | | .07 | | .49 | |
| **WR CAMIC off** | 380 | 444 | 2780 | 4323 | 1800 | 2924 | 200 | 682 |
| **t-test (vs control)** | <.001 | | .003 | | .46 | | .54 | |
| **MAMC Control (No CAMIC)** | 16713 | 3231 | 4420 | 1157 | 1720 | 655 | 783 | 186 |
| **MAMC CAMIC on** | 220 | 275 | 124 | 262 | 52 | 324 | 90 | 340 |
| **t-test (vs control)** | <.001 | | <.001 | | .003 | | .01 | |
| **MAMC CAMIC off** | 160 | 515 | 20 | 462 | -146 | 527 | -180 | 429 |
| **t-test (vs control)** | <.001 | | <.001 | | .004 | | .01 | |

**eTable 2. Particulate counts for nebulizer model.**

| All values particulate/cm3 | **1 minute** | | **3 minutes** | | **5 minutes** | | **7 minutes** | | **9 minutes** | | **11 minutes** | | **13 minutes** | |
| --- | --- | --- | --- | --- | --- | --- | --- | --- | --- | --- | --- | --- | --- | --- |
|  | **Mean** | **SD** | **Mean** | **SD** | **Mean** | **SD** | **Mean** | **SD** | **Mean** | **SD** | **Mean** | **SD** | **Mean** | **SD** |
| **WR Control (No Camic)** | 59627 | 29013 | 46960 | 35365 | 39960 | 16569 | 7393 | 2391 | 4193 | 2260 | 2427 | 2398 | 927 | 2225 |
| **WR CAMIC on** | 200 | 274 | 60 | 152 | 0 | 122 | 80 | 130 | 40 | 134 | 80 | 84 | -20 | 84 |
| **t-test (vs control)** | .003 | | .02 | | .001 | | <.001 | | .005 | | .06 | | .35 | |
| **WR CAMIC off** | 3060 | 1774 | 6120 | 3543 | 20520 | 20626 | 6820 | 12357 | 1440 | 456 | 1040 | 434 | 380 | 487 |
| **t-test (vs control)** | .004 | | .04 | | .21 | | .94 | | .03 | | .23 | | .60 | |
| **MAMC Control (No Camic)** | 122687 | 25507 | 182020 | 38028 | 214020 | 105843 | 10220 | 101 | 4903 | 326 | 2330 | 252 | 1030 | 161 |
| **MAMC CAMIC on** | 120 | 39 | 38 | 148 | 34 | 182 | -152 | 204 | -110 | 246 | -100 | 208 | -102 | 251 |
| **t-test (vs control)** | <.001 | | <.001 | | .003 | | <.001 | | <.001 | | <.001 | | <.001 | |
| **MAMC CAMIC off** | 1206 | 610 | 2546 | 1445 | 1302 | 730 | 266 | 138 | 32 | 168 | 62 | 203 | 94 | 223 |
| **t-test (vs control)** | <.001 | | <.001 | | .003 | | <.001 | | <.001 | | <.001 | | <.001 | |
